# Iron-Sulfur World in Aerobic and Hyperthermoacidophilic Archaea *Sulfolobus*

**DOI:** 10.1155/2010/842639

**Published:** 2010-09-19

**Authors:** Toshio Iwasaki

**Affiliations:** Department of Biochemistry and Molecular Biology, Nippon Medical School, Sendagi, Bunkyo-ku, Tokyo 113-8602, Japan

## Abstract

The general importance of the Fe-S cluster prosthetic groups in biology is primarily attributable to specific features of iron and sulfur chemistry, and the assembly and interplay of the Fe-S cluster core with the surrounding protein is the key to in-depth understanding of the underlying mechanisms. In the aerobic and thermoacidophilic archaea, zinc-containing ferredoxin is abundant in the cytoplasm, functioning as a key electron carrier, and many Fe-S enzymes are produced to participate in the central metabolic and energetic pathways. *De novo* formation of intracellular Fe-S clusters does not occur spontaneously but most likely requires the operation of a SufBCD complex of the SUF machinery, which is the only Fe-S cluster biosynthesis system conserved in these archaea. In this paper, a brief introduction to the buildup and maintenance of the intracellular Fe-S world in aerobic and hyperthermoacidophilic crenarchaeotes, mainly *Sulfolobus*, is given in the biochemical, genetic, and evolutionary context.

## 1. Introduction

The structure of a metal site in metalloenzymes critically influences the fine-tuning of redox and/or catalytic activities in biology [1–3], and the substitution and/or displacement events at the local metal-binding site(s) in a protein might have greatly enhanced their capabilities of conducting a wide range of unique redox chemistry in biological electron transfer conduits which often use a limited number of basic protein scaffolds. Iron-sulfur (Fe-S) cluster prosthetic groups, consisting of nonheme iron and acid-labile inorganic sulfide atoms, are functionally highly versatile and may be among the most ancient *modular* metallocofactors to sustain biologically and evolutionary indispensable processes in the early days of primitive life on earth, such as electron transfer, substrate binding/activation in the iron/sulfur storage, hydrogen and nitrogen metabolisms, anaerobic respiration, and photosynthesis—some of the most complicated reactions in the chemistry of life processes [1, 2, 4, 5]. Among protein-bound Fe-S redox sites, which usually contain terminal sulfur ligands from cysteinyl groups, the mononuclear Fe atom in a tetrahedral environment of S ligands is the simplest form, as seen in the rubredoxin family. Other major forms are polynuclear clusters, such as those containing [2Fe-2S], [3Fe-4S], [4Fe-4S], or [8Fe-7S] core units, found in a variety of ferredoxins and complex Fe-S metalloenzymes. In addition to their electron transfer roles, Fe-S proteins are also known to participate in substrate binding/activation, environmental sensing and gene regulation [2, 5–7], and more recently are suggested to be potentially involved in several human diseases [8, 9]. The physiological importance of the Fe-S clusters is largely attributable to specific features of iron and sulfur chemistry, and the biogenesis and interplay of the Fe-S cluster core with the surrounding protein is the key to in-depth understanding of the underlying mechanisms at atomic resolution.

 The archaeal domain contains organisms having the most extraordinary optimal growth conditions, with members flourishing at the extremes of pH, temperature, and salinity. The majority of thermophilic archaea are anaerobic organisms, because oxygen is often scarce in their habitats [10–13]. Biochemical and genetic evidence indicates that one of the characteristic features in the central metabolic pathways of *both* anaerobic and more unusual aerobic archaea is the involvement of small modular Fe-S proteins called ferredoxins in electron transport, and many Fe-S enzymes are produced as well in the cells [4, 14–17]. The cytoplasm of chemoheterotrophically-grown aerobic and thermoacidophilic archaea such as *Sulfolobus tokodaii* and *Thermoplasma acidophilum* is in fact remarkably enriched with bacterial-type ferredoxin(s), containing at least ~10 times more than some aerobic and thermophilic “fast-clock” bacteria such as *Thermus thermophilus* HB8 (unpublished results).

The variation of a common theme in the ferredoxin-dependent pathways of anaerobic and aerobic archaea is striking, especially considering the early days of living organisms on earth, during which the atmosphere became progressively oxidative due to the emergence of photosynthesis by cyanobacterial ancestors that leads to the prevalence of environmental iron in the trivalent state. Under these conditions *microaerobic* archaeal ancestors had to adapt to the circumstances where, in some cases, the concentration of soluble iron compounds is *below* their physiological demands. Diminishing iron levels posed a serious challenge for early *aerobic* archaea. Additionally, the polynuclear Fe-S cluster prosthetic groups contain “acid-labile” sulfide bridges, which are inherently unstable at very acidic conditions [2, 5, 18]. The stunning capability of some contemporary *aerobic and thermoacidophilic* archaea to grow at extremely low pH with intact Fe-S clusters [19–22] has implicit meaning in that the intracellular Fe-S world must be protected not only by scavenging reactive oxygen species but also by balancing their intracellular pH at an acceptable value in the face of a huge proton gradient. This short review provides a brief introduction to the buildup and maintenance of the intracellular Fe-S world in *aerobic and thermoacidophilic* archaea, mainly *Sulfolobus*. The properties of Fe-S proteins from *anaerobic and hyperthermophilic* archaea have been extensively reviewed elsewhere by others [4, 23–26].

## 2. Zinc-Containing Ferredoxins Are Abundant in the Aerobic and Thermoacidophilic Archaeal Cells

The physiological significance of bacterial-type ferredoxins in the aerobic and thermoacidophilic archaea, such as *Sulfolobus* and *Thermoplasma*, was first recognized by Kerscher et al. [15], who demonstrated that ferredoxins are abundant in the cytoplasm and serve as an effective electron acceptor of a coenzyme A-acylating 2-oxoacid:ferredoxin oxidoreductase. It is a key Fe-S enzyme of the oxidative tricarboxylic acid cycle and of coenzyme A-dependent pyruvate oxidation in aerobic archaea [15, 16, 27–30]. This oxidation takes the place in a NAD^+^-dependent 2-oxoacid dehydrogenase multienzyme complex in most aerobic and mesophilic bacteria and eukarya [15, 30]. Many 2-oxoacid:ferredoxin oxidoreductase paralogs have been identified in hyperthermophilic archaea and bacteria, some of which participate in the ferredoxin-dependent peptide fermentation pathways [24, 31]. It remains to be established how enzymatically reduced ferredoxin is reoxidized in aerobic and thermoacidophilic archaea.

 The X-ray crystal structure of the A_2_-type pyruvate:ferredoxin oxidoreductase from *Desulfovibrio africanus* has been determined by Chabrière et al. [32, 33] and shown to contain one thiamine pyrophosphate, one Mg^2+^, and three [4Fe-4S] clusters as prosthetic groups per protomer. The ab-/a_2_b_2_-type homologs from aerobic archaea inherently lack the Fe-S subunit/domain called *δ*, which harbors two [4Fe-4S] clusters [30, 34], presumably as an evolutionary consequence of one protein adaptation strategy occurring under permanently oxidative conditions. Likewise, the superfamily of archaeal and bacterial 2-oxoacid:ferredoxin oxidoreductases have different molecular sizes and subunit compositions and exhibit highly mosaic structures with respect to their domain/subunit arrangements. This implies that they might have evolved through multiple gene duplication, fusion, and reorganization events of primordial smaller fragments [17, 30, 31]. Notably, many other Fe-S enzyme complexes in biology seem to have evolved by modular evolution in an analogous way [35–38], through which the representative superfamily has become functionally divergent to meet the physiological demands.

**Figure 1:**
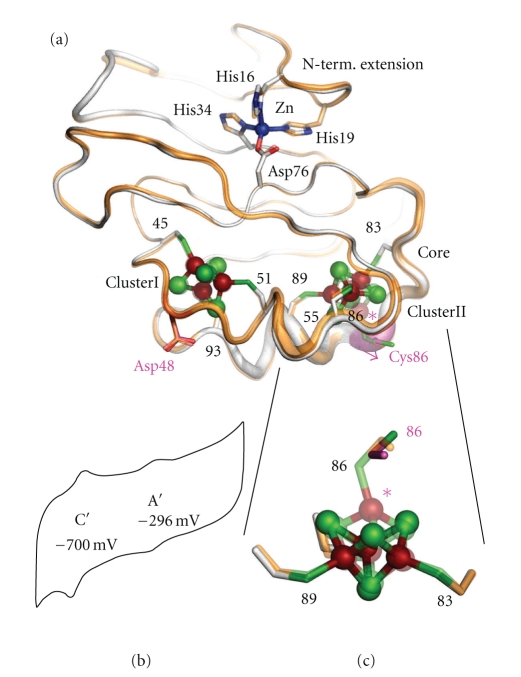
Comparative structures by superposition of archaeal zinc-containing ferredoxins from *S. tokodaii* (6Fe form, mostly gray, and pink for Asp48 and Cys86; 1xer.pdb [47, 48]) and *A. ambivalens* (7Fe form, transparent orange; 2vkr.pdb [49]), drawn in *B*-factor putty mode with *PyMOL* <http://pymol.sourceforge.net/> (a), and their close-up view by superposition of the cluster II site (c). In panels (a) and (c), key residues are labeled; pink asterisk indicates the special iron of the cluster II, which is missing in the 6Fe form (1xer.pdb). Typical fast-scan film voltammogram (at 400 mV·s^−1^) of the 6Fe form of zinc-containing ferredoxin purified from *S. tokodaii* [17, 44] (measured with a pyrolytic graphite “edge” (PGE) electrode in 5 mM each of MES/PIPES/HEPES buffer, pH 7.0, containing 100 mM NaCl and 0.2 mg/ml poly-L-lysine (Sigma) as a protomer [16]; Couple A′ (for [3Fe-4S]^1+/0^), *E*_1/2_ = −296 mV (versus NHE); Couple C′ (for [3Fe-4S]^0/2-^), *E*_1/2_ = −700 mV (versus NHE); note that wave couple B for [4Fe-4S]^2+/1+^ (*E*_1/2_ = −530 mV versus NHE) [16] was undetectable in the cyclic voltammogram) [T.I. and K. Tanaka, unpublished results] (b).

 Major ferredoxins from chemoheterotrophically-grown aerobic and thermoacidophilic archaea such as *Sulfolobus* and *Thermoplasma*, which serve as electron acceptors of 2-oxoacid:ferredoxin oxidoreductases, are characterized by relatively higher molecular masses for bacterial-type ferredoxins (~12–16 kDa) because of a long N-terminal extension region and central loop attached to the ferredoxin core fold [15, 16, 39–44]. Unlike small [4Fe-4S] ferredoxins from some anaerobic and hyperthermophilic archaea such as *Pyrococcus furiosus* [4, 25, 45] and *Thermococcus profundus* [46], they harbor one each of low-potential [3Fe-4S]^1+,0^ and [4Fe-4S]^2+,1+^ clusters. The most unusual feature of these ferredoxins is the presence of an isolated zinc center [17, 41, 43, 44, 47–49], and hence they are called the “zinc-containing ferredoxins” (Figure 1).

The isolated zinc site was first identified by the 2.00-Å structure of the *S. tokodaii* ferredoxin (PDB code, 1xer.pdb) in conjunction with the metal content analysis [41, 47, 48]. It is tetrahedrally coordinated with three histidine imidazole groups (contributed by His16, His19, and His34 in the N-terminal extension region, consisting of three *β*-strands and one *α*-helix) and one carboxylate group (contributed by Asp76 in the ferredoxin core fold). This zinc site is buried within the molecule (about 5 Å deep from the protein surface), in the boundary between the N-terminal extension and the cluster-binding ferredoxin core fold, connecting these together (Figure 1(a)). Subsequently, the zinc K-edge X-ray absorption spectroscopic analysis has shown the presence of an isolated and structurally conserved zinc center in *S. tokodaii* and *T. acidphilum* zinc-containing ferredoxins. This center is tetrahedrally coordinated with (most likely) three histidine imidazoles and one carboxylate, with the average Zn–N bond distance of 2.01 Å and the Zn–O bond distance in the range 1.89–1.94 Å [43]. These values are very similar to the average crystallographic Zn–N and Zn–O bond distance of 1.96 Å and 1.90 Å, respectively, in the *S. tokodaii* zinc-containing ferredoxin structure [47, 48]. The sequence comparisons suggest that three histidine residues in the N-terminal extension region and one conserved aspartate in the ferredoxin core fold are strictly conserved in all archaeal zinc-containing ferredoxins [17, 41, 43] (see Figure 1(a)), which suggests that they probably serve as ligands to the isolated zinc center. Although the isolated zinc site apparently contributes in part to ferredoxin thermal stability [50–52], zinc-lacking isoforms, for example, from *Sulfolobus metallicus* [53] and *Acidianus ambivalens* [54], have devised a natural strategy that accounts for an enhanced thermal stability without using the zinc site. It remains unknown whether another metal such as iron could replace the mononuclear zinc site of zinc-containing ferredoxin, when the archaeal cells are grown under zinc-limited conditions. Alternatively, ferredoxin(s) without zinc may functionally replace a zinc-containing ferredoxin under these conditions.

The overall protein fold of archaeal zinc-containing ferredoxins is largely asymmetric due to the presence of a long N-terminal extension and the insertion of central loop region (Figure 1(a)). Nevertheless, the ferredoxin core fold shows the strict conservation of a *pseudo*-two-fold symmetry with respect to the local two Fe-S cluster binding sites, as typically found for the bacterial-type 8Fe-containing dicluster ferredoxins harboring two [4Fe-4S]^2+,1+^ clusters [17, 44]. It seems reasonable to postulate that early zinc-containing ferredoxins might have evolved as an 8Fe-containing two-electron carrier without zinc, to which the N-terminal extension and central loop regions were attached in the later stage of modular evolution. Interestingly, zinc-containing ferredoxins exhibit limited distribution in the archaeal domain (such as the Sulfolobales, Halobacteriales, and Thermoplasmatales) at the genomic sequence level, and up to now have been purified exclusively from the *aerobic and thermoacidophilic* archaea such as *Sulfolobus* and *Thermoplasma* [17]. It is emphasized that, in thermophilic euryarchaeotes, zinc-containing ferredoxin has been isolated as a major ferredoxin from the Thermoplasmatales but not Halobacteriales, an unexpected result based on the universal 16S rRNA-based phylogenetic tree [41, 43]. Analogous observation has been reported for the functionally equivalent ferredoxins of extremely halophilic and aerobic euryarchaeotes [14, 55], which contain a single plant-type [2Fe-2S] cluster and exhibit the sequence similarity to those of extremely halophilic cyanobacteria [56, 57]. It should be noted that the bacterial-type and (usually more oxygen-tolerant) plant-type ferredoxins are totally unrelated at the primary and tertiary structural levels. These observations lend credence for possible phylogenetic distribution of these archaeal ferredoxin genes driven in part by horizontal (lateral) gene transfer in the extreme environments.

Biochemical and biophysical data showed that* all *archaeal native zinc-containing ferredoxins contain one [3Fe-4S]^1+,0^ cluster (cluster I) and one [4Fe-4S]^2+,1+^ cluster (cluster II) [16, 39–44]. In *S. tokodaii* zinc-containing ferredoxin, cluster I (*E*_*m*_ = −280 mV) is selectively reduced by the cognate 2-oxoacid:ferredoxin oxidoreductase during the steady-state turnover in the presence of 2-oxoglutarate and coenzyme A, while the bulk of cluster II (*E*_*m*_ = −530 mV) remains in the oxidized state [16]. This suggests that the cluster I plays a crucial redox role in the physiological electron transfer. Crystal structures of *S. tokodaii* (1xer.pdb) [47, 48] and *A. ambivalens* (2vkr.pdb) [49] zinc-containing ferredoxins indicate that the [3Fe-4S] cluster I is bound to the polypeptide chain by three cysteinyl residues, Cys45, Cys51, and Cys93 (Figure 1(a)). Residue Asp48 (a potential ligand for a fourth site, if the cluster I were a [4Fe-4S] cluster) is not bound and its carboxyl O_*δ*_1 connects to the side chain O_*γ*_ and the main chain amide NH of Ser50 by hydrogen bonds, away from the [3Fe-4S] cluster I. It should be added that [4Fe-4S] ferredoxins from anaerobic and hyperthermophilic archaea such as *P. furiosus* [25, 45] and *T. profundus* [46] contain a conserved aspartate residue at the equivalent position, serving as a ligand to the oxygen-labile [4Fe-4S]^2+.1+^ cluster. It has been reported that a one-electron reduced [3Fe-4S]^0^ cluster I of the Sulfolobales zinc-containing ferredoxins undergoes a one-proton uptake reaction, and that further two-electron hyper-reduction, which also involves uptake of protons, reversibly produces a stable, hyper-reduced [3Fe-4S]^2-^ species containing the formal equivalent of three ferrous ions [16, 39, 40, 42] (see Figure 1(b)).

 An unexpected result from the structure of *S. tokodaii* zinc-containing ferredoxin (1xer.pdb) [47, 48] was that the cluster II is converted to a cuboidal [3Fe-4S] cluster, ligated by only three cysteinyl residues, Cys55, Cys83, and Cys89, in the lattice (Figures 1(a) and 1(c)). The side chain of Cys86, a potential ligand for a fourth site, is not bound but apparently rotated toward the solvent, away from the cluster II. Additionally, the electron density for Cys86 is much lower than those of other cysteinyl ligand residues (1xer.pdb). Given the *pseudo*-two-fold symmetry of a ferredoxin core fold of bacterial-type ferredoxins, the structure indicates that, whenever a [3Fe-4S] cluster is present (and regardless of the cluster site I or II), the missing corner (Fe) of the cube is associated with either replacement (e.g., Cys^II^ → Asp, as observed for archaeal zinc-containing ferredoxins) or tilting away to the solvent of the second cysteine residue (Cys^II^) in the -Cys^I^-XaaXaa-Cys^II^-XaaXaa-Cys^III^-XaaXaaXaa-Cys^IV^-(Pro)- motif [17, 44] (Figure 1(c)). More recently, the structure of the 2.01-Å structure of *A. ambivalens* zinc-containing ferredoxin, harboring one [3Fe-4S] cluster and one [4Fe-4S] cluster, was reported (PDB code, 1vkr.pdb) [49], confirming this concept (Figures 1(a) and 1(c)).

The presence of two [3Fe-4S] clusters is very unusual in the bacterial-type dicluster ferredoxins. Biochemical and spectroscopic analyses of *S. tokodaii* zinc-containing ferredoxin showed that the 6Fe-containing species, harboring two [3Fe-4S] clusters in the lattice (Figure 1(a)), is an artifact of the crystallization procedures at pH 5; it also represents a stable intermediate produced by mild oxidative degradation of the cluster II site that occurs very slowly in solution at pH 5 *in vitro* and does not degrade Fe-S clusters to the point of an apoprotein [44]. This raises the question of how the intact Fe-S clusters of zinc-containing ferredoxins, abundant in the cells, are maintained *in vivo*, given that the intracellular pH value of aerobic and thermoacidophilic archaea is estimated around pH 5.6–6.5 [21, 58–60]. One likely possibility is that damaged Fe-S clusters are rapidly repaired *in vivo* as in *Escherichia coli* [61], but nothing is known to date about the mechanism of the archaeal Fe-S cluster repair system.

Contemporary aerobic and anaerobic archaea apparently inherited the intracellular Fe-S world from their *anaerobic* ancestors, which can be explained by the emergence of Fe-S clusters as central catalysts of metabolism from when life had evolved in an anaerobic environment. The stunning capability of some *aerobic and thermoacidophilic* archaea to grow at extremely low pH [19–22] has therefore implicit meaning in that the intracellular Fe-S world must be protected not only by scavenging reactive oxygen species (e.g., see [62–67]) but also by balancing the intracellular pH at an acceptable value (typically 5.6–6.5 in *Sulfolobus* and *Thermoplasma* [21, 58–60]) in the face of a huge proton gradient (∆pH = pH_in_ − pH_out_). The ∆pH across the cytoplasmic membrane of these archaea is intrinsically linked to the cellular energetics [21, 58, 68], because it is the primary contributor to the proton motive force (PMF)

(1) $$\text{PMF}=∆\Psi -2.3\left(\frac{\text{RT}}{\text{F}}\right)∆\text{pH}\;\;\left(\text{mV}\right),$$

where ∆Ψ is the membrane potential generated by the transport of electrical charge, R the gas constant, T the absolute temperature (K), and F the Faraday constant (the effect of 1 unit pH difference is 2.3(RT/F), which equals 59 mV at 25°C and 70 mV at 80°C). However, the influx of protons through the archaeal *A*_*o*_ *A*_1_-ATP synthase upon ATP production [22, 60, 69] intensifies cellular protonation, and therefore need to be balanced by extrusion using the cognate proton translocating systems to remove excess protons from the cytoplasm (otherwise, this would simply result in rapid acidification of the cytoplasm and dissipate any ∆pH formation across the membrane [21, 58–60, 68]). Thus, the balance between the proton permeability across the membrane (kept *very low* in thermoacidoacidophiles), the proton influx through the energetic and transport systems, and the rate of outward proton pumping determines whether an archaeal cell can sustain an appropriate PMF. The mechanistic detail of how this thin-line balance could be achieved has not been clearly understood.

A mechanism used by some acidophilic archaea such as *Thermoplasma* to reduce the proton influx is the generation of an inside positive ∆Ψ [59], which is opposite to the inside negative ∆Ψ in mammalian mitochondria. It is suggested that the reversed ∆Ψ is generated by a difference in electrical potential (Donnan potential) formed between a greater influx of cations (such as potassium ions) and the outward flux of protons [19, 21, 59]. This is in line with our preliminary study on the aerobic respiratory chain of *T. acidophilum*, which contains at least cytochrome *bd* as a terminal oxidase (unpublished results) that is usually not a proton pump. However, this concept is apparently not applicable to *Sulfolobus*, where the inside negative ∆Ψ is rather low (about −20 to −40 mV at 45°C) and the PMF is largely composed of a ∆pH of greater than 2 units [58, 60].

As reviewed elsewhere [68, 70–75], the *Sulfolobus* species have the unusual terminal oxidase segments of the aerobic respiratory chain, consisting mainly of only *a*- and *b*-type cytochromes, which most likely fulfill the role as an effective proton pump *in vivo* and preserve the cognate Fe-S world. Primary dehydrogenases, some of which are complex Fe-S enzymes, provide the reducing equivalents (from the respiratory substrates such as succinate, NADH, and sulfide) to the central caldariellaquinone pool in the tetraetherlipid membrane [35, 68, 71, 74, 75]. It should be commented here; however, that most of key biochemical/genetic characterization of the *Sulfolobus* respiratory chains (e.g., [68, 71, 72, 76–78]) were carried out before the availability of a variety of the genome-wide sequence information [74, 75, 79, 80] and the mechanistically insightful 3D structures of the terminal segments of the tractable respiratory complexes from bacteria and eukaryal mitochondria (reviewed in [81–87]). In retrospect, many experimental data in the literature from the pregenomic era were discussed in seemingly oversimplified ways, perhaps inspired by an idea that an archaeal aerobic respiratory chain might be “primitive and simple”. The archaeal respiratory chains may be in fact archaic, but not so primitive as they had seemed two decades ago. For instance, the *S. tokodaii* genomic sequence [74] shows at least seven paralogous genes coding for the putative quinol/cytochrome oxidase subunit I superfamily, two of which are homologs of SoxB [76] and SoxM [77] of *S. acidocaldarius*; of course, not all of these proteins may be spontaneously expressed to function as true respiratory terminal oxidases (some of them may be induced only under specific growth conditions [88–90] and/or serve as a putative oxygen sensor(s) for aerotaxis [91]). Additionally, while the terminal oxidase supercomplexes of *S. acidocaldarius* (SoxABCD and SoxM supercomplexes [68, 76, 77]) and *S. tokodaii* [71] (presumably SoxABCD-like supercomplex as estimated from the similarity of the redox potentials of heme A_S_ centers [68]) have been shown to mimic a genetic and functional fusion of mitochondrial respiratory complexes III and IV, the number of the redox centers resolved spectroscopically in the literature is insufficient to explain the proposed intramolecular electron transfer mechanism, particularly in the light of a modified Q-cycle scheme of respiratory complex III, which is characterized by bifurcation of electron transfer between two different acceptor chains that allows coupling to proton transfer [85–87, 92]. Thus, the *Sulfolobus* aerobic respiratory chain in a mechanistic context is still in its infancy compared with the mitochondrial and bacterial tractable model systems, and needs to be explored in future studies.

 In the thermoacidophilic archaea, the transmembrane ∆pH-driven secondary transporters for peptides, sugars, and inorganic compounds are preferred over primary (ABC) transporter systems [19–21], which is not surprising given a permanent huge ∆pH across the membrane. Available genomic sequences of the *Sulfolobus* species [74, 75, 79, 93] suggest the presence of metal transporter homologs [20, 22, 94, 95], some of which may be involved in trafficking iron ions for the biogenesis of Fe-S proteins. Very little is known to date about *in vivo* iron-trafficking and homeostasis systems in these archaea (e.g., [88–90]), and further genome-wide “omics” approaches in a functional context may bring a better understanding of these mechanisms.

## 3. Formation of Intracellular Fe-S Clusters Does Not Occur Spontaneously but Requires Specific Biosynthetic Pathways

In contemporary bacteria and eukarya, the *de novo* Fe-S cluster biogenesis and maturation* in vivo* have been shown to require specific enzymes in the carefully regulated Fe-S cluster biosynthesis systems [5, 7–9, 96–101], while spontaneous assembly of the Fe-S clusters does occur *in vitro*. At least three types of the Fe-S cluster biosynthesis systems (ISC (*i*ron *s*ulfur *c*luster), SUF (mobilization of *su*l*f*ur), and NIF (*ni*trogen *f*ixation)) are known, with significant variations in terms of the phylogenetic distribution [7, 99–101]. For example, in *Escherichia coli* the ISC pathway [102–104] is the major system for *in vivo* Fe-S cluster assembly, compared to the SUF pathway [98, 105]. In the eukaryal domain [7, 8], ISC homologs are found to be localized largely in mitochondria, while SUF homologs are found in some chloroplasts. It is therefore possible to postulate that the mitochondrial ISC system originated from the endosymbiotic bacterial ancestor and the plastid SUF system from the cyanobacterial ancestor. In these tractable model organisms, the regulation of biological Fe-S cluster assembly is further complicated by the involvement of other accessory proteins required for the *in vivo* function [7, 8, 99, 101, 106, 107], and is not fully understood.

**Figure 2:**
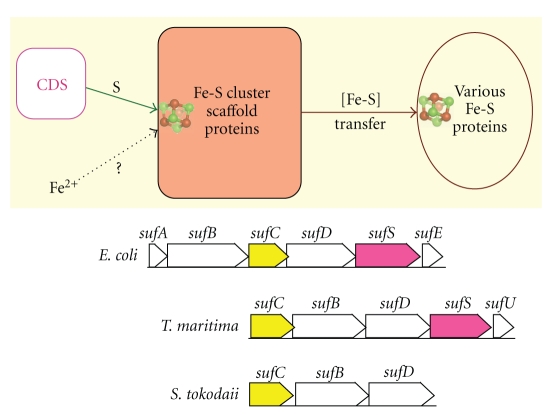
Schematic illustration of the cysteine desulfurase (CDS)-mediated, transient Fe-S cluster assembly on Fe-S cluster scaffold proteins and subsequent cluster transfer to various target apoproteins [7, 99, 116] (top), and the organization of the *suf *gene clusters annotated in the *E. coli*, *T. maritima*, and *S. tokodaii* genomic sequences (bottom).

The common concept of the three *de novo* Fe-S cluster biosynthesis systems is that *in vivo* cluster assembly requires at least (i) cysteine desulfurases (such as NifS, IscS and SufS) [105, 108–113] and (ii) Fe-S cluster scaffold proteins (such as IscU, IscA, SufU, and likely SufBCD) with the capacity to construct transient [2Fe-2S] or [4Fe-4S] clusters and then transfer Fe-S clusters to target apo-proteins [114–120] (as schematically illustrated in Figure 2). While pyridoxal phosphate-containing cysteine desulfurases utilize L-cysteine for mobilization of S for Fe-S core formation, there is as yet no consensus concerning immediate iron donor for Fe-S cluster assembly. The ISC machinery has been most closely investigated, and bacterial genomic sequence analyses showed the relatively conserved gene arrangement as *iscR*-*iscS*-*iscU*-*iscA*-*hscB*-*hscA*-*fdx* [102, 109, 121], where IscR is a transcriptional regulatory protein, HscA/HscB DnaK/J-type heat-shock proteins, and Fdx an adrenodoxin-like [2Fe-2S] ferredoxin.

The importance of the SUF machinery in the Fe-S cluster biosynthesis function was clarified in *E. coli* by construction of different combinations for altered expression of the ISC and SUF operons [98, 122, 123]. Disruption of the *E. coli suf* operon does not cause any major defects, whereas the loss of both the ISC and SUF systems leads to synthetic lethality. The components of the *suf* operon has been shown to be preferred for Fe-S cluster biosnthesis under oxidative stress conditions [124, 125] and during iron starvation [122] although the ISC and SUF systems are principally interexchangeable, especially in an anaerobic environment [123]. In *E. coli* and *Thermotoga maritima*, the *suf* gene cluster is arranged as* sufA-sufB-sufC-sufD-sufS-sufE1* and *sufC-sufB-sufD-sufS-sufU*, respectively, [98, 105] (Figure 2, bottom). In some hyperthermophilic archaea and bacteria, the SUF system has been proposed to be the sole pathway for cluster assembly [98, 126]. This implies that some components of the hyperthermophile SUF-related system might represent a primordial pathway for the Fe-S cluster biogenesis.

Although aerobic and anaerobic archaea produce numerous Fe-S proteins, the major components of the bacterial and eukaryal Fe-S cluster biosynthesis systems are not universally conserved in archaea. In *S. tokodaii* [74] (and some other archaeal species), only the *sufB*, *sufC*, and *sufD* genes are conserved, which are arranged as the *sufC*(ST1201)-*sufB*(ST1200)-*sufD*(ST1199) gene cluster (Figure 2, bottom). SufB and SufD are paralogs and form a water-soluble, unorthodox ATP-binding cassette-like complex with SufC that has intrinsic ATPase activity [113, 127]. No *sufA* homolog could be identified in this archaeal genomic sequence [74, 126]. This is in line with a detection of archaeal SufBCD complex by the native proteome approach from native biomass using *P. furiosus* [128]. Recently, the *E. coli* SufBC_(2)_D complex has been shown to function as a novel Fe-S scaffold machine and interacts with SufA for the Fe-S cluster transfer [119, 129], and formation of the oxygen-labile [4Fe-4S] cluster was characterized by *in vitro* reconstitution of SufBC_2_D under anaerobic conditions [120]. These findings strongly argue for the archaeal SufBC_(2)_D complex functioning as a possible Fe-S scaffold machine.

While SufA is absent in most archaea [126] (Figure 2, bottom), the homologs of the bacterial *apbC* [130, 131] and eukaryotic *NBP35* [107, 132] genes, coding for Fe-S cluster carrier proteins, are conserved in some archaea [133] (ST0174 in the *S. tokodaii* genomic sequence [74]). ApbC from *Salmonella enterica* is a homodimeric ATPase which can bind an Fe-S (presumably [4Fe-4S]) cluster and activate yeast apo-isopropylmalate dehydratase (apo-Leu1) *in vitro*, in an ATP-independent manner [130, 131], and the *S. enterica* strains defective in *apbC* (*mrp* in *E. coli*) showed that alterned thiamine biosynthesis are impaired in Fe-S cluster metabolism [134]. Likewise, the eukaryal ApbC homologs Cfd1 and Nbp35 form the extramitochondrial homotetrameric complex, and bind labile [4Fe-4S] clusters (after *in vitro* reconstitution), which can be transferred to target Fe-S apoproteins but only when other CIA (*c*ytosolic *i*ron-sulfur protein *a*ssembly) proteins Nar1 and Cia1 co-exist [107]. The archaeal ApbC/NBP35 homolog shows similar properties as *S. enterica* ApbC [133], and is a putative candidate for an Fe-S cluster shuttle that delivers a preassembled Fe-S cluster to a recipient apoprotein, although nothing is known to date about its interplay with the cognate SUF system.

A missing piece in the SUF system of aerobic and thermoacidophilic archaea is a cysteine desulfurase (IscS/SufS/CsdA) homolog (see Figure 2, bottom). For example, an archaeal SufS homolog was recently identified from *Haloferax volcanii* [135] and a possible CsdA (but not SufS) homolog is found in the genomic sequence of* Aeropyrum pernix* K1 (APE2023 [136]), but they are poorly conserved in *S. tokodaii* (presumably ST2140, tentatively annotated as a hypothetical isopenicillin *N* epimerase [74]). Thus, an alternative possibility is still open for novel cysteine desulfurases in these archaeal SUF systems. There are very few genetic and biochemical studies (e.g., [128, 133]) on the archaeal Fe-S cluster biosynthesis system so far, and further development of the genetic manipulation systems is needed to verify these hypotheses in a functional context.

## 4. Geometric Tolerance of the Cluster Binding Loop Region and the Thiophilicity with Iron Ions Respect to the Fe-S Cluster Recognition

As described briefly in the preceding section, the *de novo* Fe-S cluster biosynthesis, which is catalyzed and regulated by a number of specific enzymes, can be divided into two major steps (Figure 2). The first step is a transient *de novo* Fe-S cluster assembly on a scaffold protein requiring sulfur and iron donors. In the second step, the transient Fe-S cluster is dislocated from the scaffold protein, followed by transfer and insertion into recipient apoproteins, either during or shortly after the apoprotein generation and before the folding into its native-like conformation. A question of how the required (and rather ill-defined) binding site of a recipient protein-matrix, often categorized as the “binding motif” in the genome-wide bioinformatics, could select and bind a specific Fe-S cluster in the Fe-S protein biogenesis is considered in this section.

**Figure 3:**
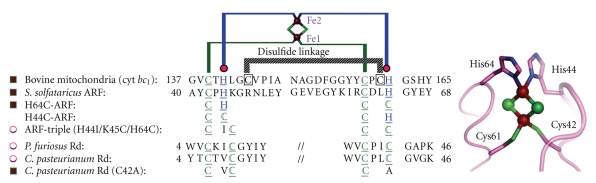
Multiple sequence alignment of the metal-binding sites of selected Rieske-type proteins and rubredoxins (Rd). The cluster-binding motif of *S. solfataricus* ARF is characteristic of Rieske-type ferredoxins involved in bacterial multicomponent oxygenases, containing two histidyl (blue) and two cysteinyl (green) ligands, and lacks two conserved cysteine residues (black) that serve as the disulfide linkage in high potential, respiratory Rieske proteins [138, 153]. DDBJ-EMBL-GenBank accession codes: bovine mitochondrial cytochrome *bc_1_*-associated Rieske protein fragment, P13272; *S. solfataricus* ARF (hypothetical ORF c06009), CAA669492, AB047031; *P. furiosus* rubredoxin, P24297; *C. pasteurianum* rubredoxin, P00268. The metal-binding motifs are underlined (left), and the structure of the cluster ligand residues of a bovine mitochondrial Rieske protein domain fragment (PDB code, 1rie.pdb) [145] is shown, but with the *S. solfataricus* ARF numbering (right). Brown square symbols (left), wild-type or mutant proteins containing a [2Fe-2S] cluster; magenta open circles (left), wild-type or mutant proteins containing a Rd-like, mononuclear (Fe/Zn) center.

Our group used an archaeal Rieske-type [2Fe-2S] ferredoxin (called ARF) from *Sulfolobus solfataricus* P1 [137–142] as a tractable model (Figure 3). Rieske-type [2Fe-2S] clusters are ubiquitous in a variety of organisms, playing crucial electron transfer functions in respiratory chains, photosynthetic chains, and multicomponent oxygenase systems for biodegradation of aromatic and alkene compounds [85, 143, 144]. In contrast to regular plant- and vertebrate-type [2Fe-2S] ferredoxins having complete cysteinyl ligations, the Rieske-type cluster has an asymmetric [2Fe-2S] core with the S_*γ*_ atom of each of the two cysteine residues coordinated to one iron site and the N_*δ*_ atom of each of the two histidine residues coordinated to the other iron site (e.g., PDB codes, 1rie, 1rfs, 1ndo, 1fqt, 1jm1, 1nyk and 2nuk.pdb [145–151]) (Figure 3, right). The structure of a bovine mitochondrial Rieske protein domain fragment suggests that its cluster-binding loops have a similar geometry to those found in the rubredoxin and zinc ribbon scaffolds [145]. We have addressed the influence of substitution of each of the two outermost histidine ligands (His44 and His64) by cysteine on the properties of the Rieske-type [2Fe-2S] cluster in *S. solfataricus* ARF (Figure 3). Replacement of one of the histidine ligands, His64, by cysteine allowed the assembly of a new low-potential [2Fe-2S] cluster with one-hisitidine plus three-cysteine ligands in the archaeal Rieske-type protein scaffold whereas replacement of the other ligand, His44, by cysteine generated a protein that failed in cluster insertion and/or assembly [138]. Replacement of the two histidine ligands to the [2Fe-2S] cluster of *S. solfataricus* ARF by cysteine residues (in the H44C/H64C double mutant) largely impaired the cluster assembly in the recombinant variant protein. In contrast, replacement of three residues (His-44, Lys-45, and His-64) in ARF by cysteines and isoleucine (H44I/K45C/H64C triple mutant), to mimic the mononuclear Fe(Cys)_4_ site in the *P. furiosus* rubredoxin [152], has allowed a rational design of the thermostable rubredoxin-like, mononuclear Fe(Cys)_4_ site in the recombinant ARF-triple mutant protein [153] (Figure 3, left).

 These experiments demonstrate that the *in vivo* assembly of a [2Fe-2S] cluster in the Rieske protein scaffold is determined primarily by the nature and spacing of the ligands at the cluster binding loops which are often located near the protein surface in modular Fe-S proteins [138, 153] (Figure 3). The two innermost cysteinyl ligand residues (Cys42 and Cys61) of *S. solfataricus* ARF are also essential for the cluster assembly and/or stability [138], suggesting that the thiophilicity of iron ions with the thiol-containing loop region is also important for the Fe-S cluster binding and/or stability. It seems plausible that a (kinetic) “native-like” semiordered structure of the cluster binding site in a folding intermediate may behave as a substrate in the enzyme-assisted [2Fe-2S] cluster assembly/maturation steps, where (i) the geometric tolerance of the metal-binding loops, allowed by the spacing and types of ligands near the protein surface, and (ii) the thiophilicity of iron ions with the thiol-containing loops should play decisive roles [153]. This is in accord with the previous report by Meyer et al. [154], clearly showing the (unexpected) assembly of an oxidized [2Fe-2S] cluster into a recombinant, single-ligand-substituted (C42A) variant of *Clostridium pasteurianum* rubredoxin, whose polypeptide chain normally accommodates a mononuclear Fe(Cys)_4_ site in the wild-type protein (see Figure 3, left).

 Although not experimentally tested, generality of this “geometrical tolerance plus thiophilicity” concept seems to also apply to the biogenesis of a cubane [4Fe-4S] cluster, considering also the established interconversion of the Fe-S cluster types (two [2Fe-2S] ↔ one [4Fe-4S]) on the IscU scaffold protein [114, 116]. Here the minimal requirement for the number of terminal cysteinyl ligands to a cubane [4Fe-4S] cluster is usually three in most simple and complex Fe-S proteins, and the fourth ligand at a (spatially) particular position can be an external ligand [2] (e.g., see Figure 1(c)). This may be the reason why a cubane [4Fe-4S] core is often employed for the substrate binding/activation in some Fe-S enzymes, such as aconitase and related hydratases, and the radical *S*-adenosylmethionine (SAM) superfamily [5, 6, 155, 156].

A likely biological and evolutionary benefit of having a polynuclear cluster site in a complex metalloenzyme would be that the cluster synthesis/assembly can be more strictly controlled by one or more specific synthesis-and-assembly apparatuses [5, 96–98], thereby facilitating a unique redox chemistry for specific cellular needs—simple binding of a mononuclear transient metal site in a primordial metalloprotein might have been more severely influenced by the *in vivo* availability of environmental metal ions to the last universal common ancestors (due to the simpler metal binding equilibrium). Additionally, a cavity of sufficiently large size to accommodate a polynuclear cluster might reduce a potential problem of binding the wrong metal ion that is correlated with the Irving-Williams series [157] of the stability trend for aqueous metal-sulfur complexes in the order, Mn^2+^< Fe^2+^< Co^2+^< Ni^2+^< Cu^2+^> Zn^2+^ (even when diminishing iron levels posed a serious challenge for early aerobic archaea). Prototypal polynuclear cluster formations, followed by early modular evolutionary events afforded “stepwise” development of new catalytic and electron transfer functions of primordial complex metalloenzymes. These enzymes consist of ensembles of redox protein modules of convergent/divergent evolutionary origins, using a limited number of basic protein scaffolds, and could meet versatile requirements of early metabolisms and environmental conditions [153]. Contemporary aerobic and thermoacidophilic archaea inherited the resultant intracellular Fe-S world from their anaerobic ancestors, and this world keeps running in an extraordinary environment by powering the enzyme-assisted Fe-S cluster biogenesis machinery.

## 5. Conclusion

The majority of thermophilic archaea are anaerobic organisms because molecular oxygen is often scarce in their habitats. Early biochemical evidence has established that one of the characteristic features in the central metabolic pathways of *both* anaerobic and aerobic archaea is the involvement of ferredoxins in electron transport. In the aerobic and thermoacidophilic archaea, zinc-containing ferredoxin [17] is abundant in the cytoplasm and functions as a key electron carrier; in addition, many other Fe-S enzymes are operative in the central metabolic and bioenergetic pathways [17, 35, 68]. These Fe-S proteins must be protected by keeping intracellular pH at an acceptable value (typically 5.6–6.5 in *Sulfolobus* and *Thermoplasma* [20, 21, 58–60]) in the face of a huge proton gradient ∆pH across the membrane. Thus, in addition to expected structural adaptations of a local Fe-S cluster binding site by natural selection, the Fe-S enzymes of aerobic and thermoacidophilic archaea obligately require the stringent intracellular pH homeostasis mechanism, as well as the reactive oxygen species-scavenging system. Some thermoacidophilic archaea such as *Thermoplasma* do this by reducing the proton influx by the generation of an inside positive membrane potential ∆Ψ, which is generated by a difference in electrical potential formed between a greater influx of cations (such as potassium ions) and the outward flux of protons [19, 21, 59]. In *Sulfolobus*, the inside negative ∆Ψ is rather low and the PMF is largely composed of a ∆pH of greater than 2 units [21, 58, 60, 68], where the cognate aerobic respiratory chain probably fulfills the role as an effective proton pump *in vivo* and preserves the cognate Fe-S world descendant from their anaerobic ancestors.

*De novo* formation of intracellular Fe-S clusters does not occur spontaneously but requires specific biosynthetic pathways: of three types of the Fe-S cluster biosynthesis systems (NIF, ISC, and SUF) identified in the bacterial and eukaryal systems [7, 98–101], the thermoacidophilic archaea apparently contain only the SUF system. More specifically, only the SufB, SufC, and SufD homologs are conserved in some archaea including *Sulfolobus*, which most likely function as a putative Fe-S scaffold complex [119, 120]. On the other hand, cysteine desulfurase (CdsA/IscS/SufS) homologs are rather poorly conserved in these archaea, and remain to be assigned in future study. A transient Fe-S cluster dislocated from the archaeal SUF scaffold protein is subsequently transferred (presumably using an ApbC/NBP35 homolog) and inserted into recipient apoproteins, either during or shortly after the apoprotein generation and before the folding into its native-like conformation. In many recipient Fe-S protein modules, the Fe-S cluster is assembled to loop regions and is often located near the protein surface. The *in vivo* assembly of a biological Fe-S cluster in a (recipient) protein scaffold is determined primarily by the nature and spacing of the ligands in the cluster binding loops. These loops probably define the geometric tolerance and thiophilicity of iron ions and thereby play a decisive role in a (kinetic) “native-like” semiordered folding intermediate. I hope that this short review will stimulate further research work, through which the answers to many open questions will be integrated into a comprehensive view on the biogenesis and maintenance of the archaeal Fe-S world.
